# A Wireless Neuroprosthesis for Patients with Drug-refractory Epilepsy: A Proof-of-Concept Study

**DOI:** 10.7759/cureus.5868

**Published:** 2019-10-09

**Authors:** Pantaleo Romanelli, Taufik A Valiante, Stefano Seri, Cosimo Puttilli, Mauro Picciafuoco, Martin Jakobs, Andres Lozano

**Affiliations:** 1 Neurosurgery, Cyberknife Center, Centro Diagnostico Italiano, Milano, ITA; 2 Neurosurgery, University of Toronto, Toronto, CAN; 3 Clinical Neurophysiology, Aston University, Birmingham, GBR; 4 Research and Development, AB Medica, Milano, ITA

**Keywords:** cortical stimulation, brain mapping, epilepsy, wireless, neuroprosthesis, electrocorticography, brain-computer interface

## Abstract

Objective

Acute or protracted cortical recording may be necessary for patients with drug-refractory epilepsy to identify the ictogenic regions before undergoing resection. Currently, these invasive recording techniques present certain limitations, one of which is the need for cables connecting the recording electrodes placed in the intracranial space with external devices displaying the recorded electrocorticographic signals. This equates to a direct connection between the sterile intracranial space with the non-sterile environment. Due to the increasing likelihood of infections with time, subdural grids are typically removed a few days after implantation, a limiting factor in localizing the epileptogenic zone if seizures are not frequent enough to be captured within this time-frame. Furthermore, patients are bound to stay in the hospital, connected by the wires to the recording device, thus increasing substantially the treatment costs. To address some of the current shortcomings of invasive monitoring, we developed a neuroprosthesis made of a subdural silicone grid connected to a wireless transmitter allowing prolonged electrocorticografic recording and direct cortical stimulation. This device consists of a silicone grid with 128-platinum/iridium contacts, connected to an implantable case providing wireless recording and stimulation. The case also houses a wirelessly rechargeable battery for chronic long-term implants. We report the results of the first human proof-of-concept trial for wireless transmission of electrocorticographic recordings using a device suited for long-term implantation in three patients with drug-refractory epilepsy.

Methods

Three patients with medically refractory epilepsy underwent the temporary intraoperative placement of the subdural grid connected to the wireless device for recording and transmission of electrocorticographic signals for a duration of five minutes before the conventional recording electrodes were placed or the ictal foci were resected.

Results

Wireless transmission of brain signals was successfully achieved. The wireless electrocorticographic signal was judged of excellent quality by a blinded neurophysiologist.

Conclusions

This preliminary experience reports the first successful placement of a wireless electrocorticographic recording device in humans. Long-term placement for prolonged wireless electrocorticographic recording in epilepsy patients will be the next step.

## Introduction

Electrocorticography (ECoG) is a technique that allows the direct recording of brain electrical activity from the cerebral cortex. Developed by Penfield and Jasper in the 1940, its use spans from guiding resection of epileptogenic tissue in patients with drug-refractory epilepsy to direct mapping of regions of the brain associated with essential functions (eloquent cortex) such as movement, sensation, language comprehension and production in response to carefully tailored stimuli. ECoG contacts can also allow direct electrical stimulation (DCS) of the human brain, which represents the standard invasive method for cortical mapping in the context of the resective brain surgery for brain tumors or other lesions encroaching eloquent cortex [1]. Mapping can be performed outside the surgical theatre with the patient fully awake, avoiding the interference of anesthetic drugs on brain activity. Recently, the use of the ECoG signal has also shown promising results in providing guide and feedback to robotic prostheses or exoskeletons aiming to restore the mobility of patients who have lost sensorimotor function due to conditions like spinal cord injury or brachial plexus [2-3]. Commercially available ECoG systems are based on a grid-array of platinum or stainless steel electrodes embedded into one or more sheets of a silastic material connected with an external amplifier through a shielded cable. The cables leave the skull through a subcutaneous tunnel, thus providing a potential path for cerebrospinal fluid (CSF) leak and consequent meningeal infection [4-6]. Infection is in fact the most common complication related to the placement of subdural grids, especially if the monitoring is extended beyond 10 days [6] . Due to the relationship between risk of infection and recording duration, ECoG recording sessions are typically limited to one week. Furthermore, patients undergoing invasive monitoring for epilepsy are confined into a dedicated recording room under careful but necessarily short observation. At the end of the monitoring period, the wound is re-opened and the grid removed. The relatively short duration of most ECoG recording sessions can result in non-conclusive findings if not enough representative seizures are captured. This limits a priori the number of patients to whom ECoG can be offered based on seizure frequency or required medication tapering to facilitate seizure occurrence. Based on these considerations, the use of cables connecting the epicortical grid to an external device appears to be the most significant shortcoming of current ECoG techniques. To address the above limitations, after a preliminary experience on primates, we developed wireless fully implantable ECoG device characterized by 128 platinum-iridium contacts placed on a silicon grid of sub-millimetric thickness and connected by intracranial cables to a case made of polyetheretherketone (PEEK) allowing wireless transmission [3,7-8]. This device allows DCS through a set of 64 dedicated electrodes, while the other 64 electrodes are used for signal acquisition only. The unique features of this neuroprosthesis include 1) wireless data transmission of all the 64 acquisition channels recording in parallel, 2) wireless direct cortical stimulation, and 3) wireless recharge by electromagnetic induction. The device has been granted the following international patents: EP2699145 B1; US9031657 B2; JP6082942 B2; CA2832520 A; AU2012245942 B2; CN103648367 B. Data transmission exploits the recently introduced Medical Implant Communication System (MICS), a low-power, short-range (2 m), high-data-rate, 401-406 MHz (the core band is 402-405 104 MHz) communication network that has been accepted worldwide for transmitting data to support the diagnostic or therapeutic functions associated with medical implant devices [9]. This novel device was designed to allow prolonged ECoG recording during invasive monitoring for several weeks/months, with possibility for the patient to be discharged and safely monitored in its home environment. ECoG signals can be monitored real time by the involved physician and personnel, embodying a novel development of telemedicine. The device can in theory also be used for a close-loop responsive stimulation and for a wide range of brain computer interface (BCI) clinical applications. Wireless ECoG acquisition using this new technique offers several clinical advantages over conventional recording techniques: 1) the risk of CSF leaks and intracranial infections is reduced; 2) prolonged recordings can be obtained; 3) remote home monitoring with the patient discharged is possible. Prolonged recordings during regular daily life can provide not only evidence of epileptic activity not shown by short-term monitoring, but also show long-term seizure patterns not otherwise visible.

## Materials and methods

Patients

This study was conducted in patients scheduled for craniotomy surgery for their epilepsy. This study was reviewed by the Surgical Innovations Committee of the University Health Network of Toronto University and approval was obtained to investigate three patients. Patients gave written informed consent to participate in the study. This study consisted of placing the wireless transmitting grid on the surface of the cortex and wirelessly recording and transmitting the ECoG for approximately five minutes. After this period, the wireless grid was removed, and the operation proceeded as planned.

The Wireless Device ECoGI-64D 126

**Figure 1:**
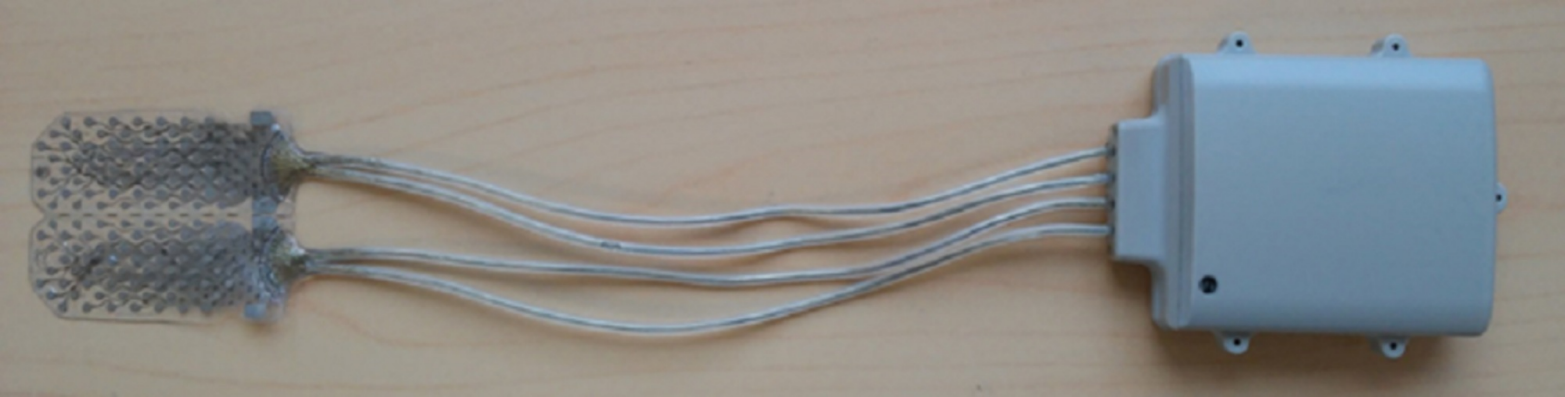
ECoGI-64D - the picture shows the subdural grid, connecting cables and a wireless case

The “ECoGI-64D System” is an implantable brain diagnostic/stimulating device designed by AB Medica to localize and monitor the activity of seizure foci in daily life for a prolonged time (up to 30 days, with continuous recording for 24 hours a day) and to map the functional activity of the brain by DCS before resection (Figure 1)*.*

**Figure 2:**
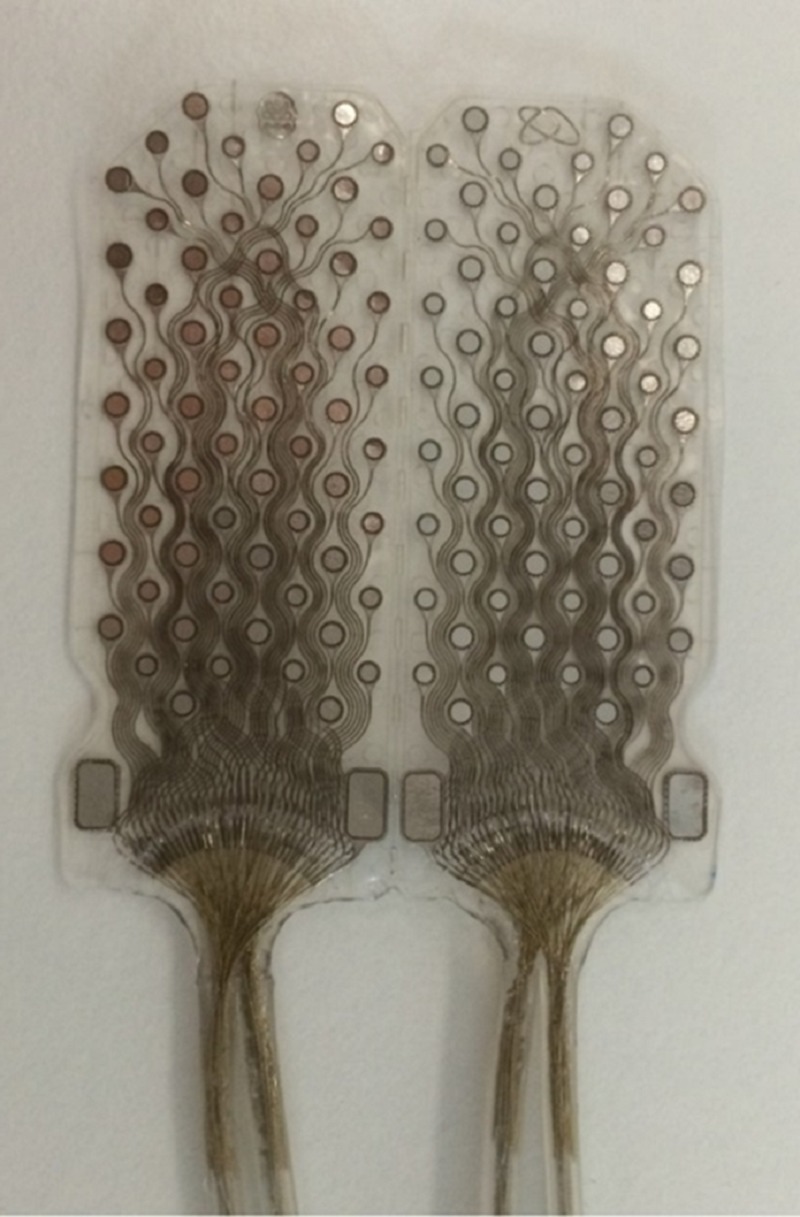
The 128 contacts subdural grid hosting 64 recording and 64 stimulating contacts plus four ground contacts According to clinical needs, various sizes can be made. The grid described here measures 43 by 36 mm.

ECoG data are acquired from the cortical surface of the patient brain and transmitted to a radio base station; an external computer with dedicated software provides control of data acquisition and visualization. The external computer has also dedicated audio/video hardware and software used to provide a synchronized video ECoG. The ECoGI-64D grid is composed of 64 acquisition and 64 stimulation electrodes (plus four reference electrodes), arranged in two separable sections (Figure 2).

The grid can be split into two symmetric parts with 32 acquisition and 32 stimulation contacts each plus two reference contacts. Electrodes are connected to an electronic board placed in an airtight case housed in a compact biocompatible sealed PEEK enclosure. The electronic board allows control, acquisition, and data communication. The antenna for communication, the coil for wireless recharge and the battery for power supply are also placed inside the airtight case. The electronic board contains an Analog to Digital front-end and a multiplexing module allowing to choose the stimulation electrodes pair and a microcontroller for device management. A radio link module sends the data to the external unit (Radio Base Station) by MICS radio signals (Medical Implant Communication Service-MICS: 402 to 405 MHz band). The microcontroller software can be upgraded remotely, providing further flexibility to the system. Wireless recharge is provided by magnetic induction through a dedicated device according to the international WPC (Wireless Power Consortium) standard. Overheating during the recharge procedure is prevented by the ECoG proprietary algorithm, which receives continuous feedback about the skin temperature above the PEEK case. The device is compliant with all the requirements of European Directives for Active Implantable Medical Devices, applicable to the cortical recording for pre-surgical evaluation of epilepsy in humans, considering all the constraints of an implantable medical device like ultra-low power, miniaturization, safety, reliability and regulatory requirements applicable. Further technical details are described in previously published papers [3,7-8]. 

Evaluation of the Intracranial Electroencephalogram

The acquired signal was submitted for blind review to a specialist clinical neurophysiologist (SS) who evaluated the suitability of the data for the clinical interpretation and for localization of interictal epileptiform activity. The latter was compared with that identified in the dataset acquired with a conventional intracranial grid.

## Results

**Figure 3:**
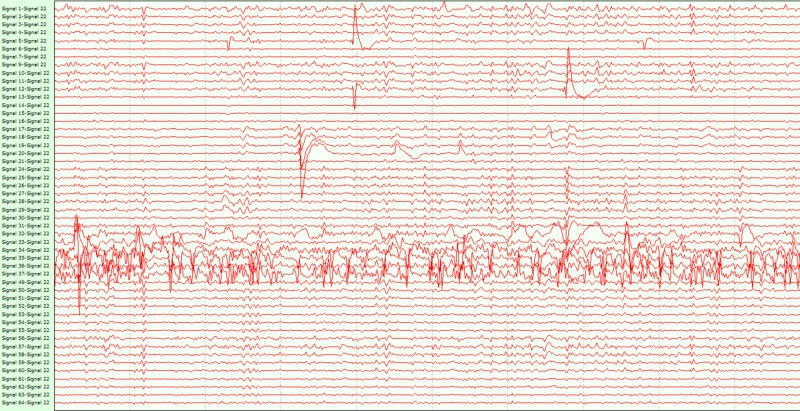
Wireless ECoG screenshot showing interictal spikes over the channels 35-37 (covering the inferior temporal gyrus) in patient 1

Short-term wireless ECoG recordings were performed in three patients during the resective epilepsy surgical procedures. Patient 1 had seizure onset at the age of 10 years. A left temporal arachnoid cyst was detected and drained, after which the patient remained seizure-free for eight years. At the age of 24 years, due to recurrent drug-refractory seizures, a left anterior temporal lobectomy was performed but seizure control was not achieved. At the time of referral, the patient was on levetiracetam, lamotrigine, and methylphenidate. Scalp video-electroencephalogram (EEG) telemetry showed ictal activity in the left posterior quadrant. To precisely outline the extent of the epileptogenic zone, the patient underwent an extension of a previous craniotomy under general anesthesia and the EcoG recording. The dura was opened, and the prototype grid was placed on the temporal neocortex, the basal temporal neocortex, and the superior temporal gyrus. Interictal ECoG signals were recorded and wirelessly transmitted for five minutes. Figure 3 shows a screenshot of the wireless ECoG with interictal epileptic activity from this patient. After the wireless recording was completed, the device was removed and replaced by a conventional grid for diagnostic invasive monitoring. A week later, the patient underwent removal of the implanted grid and microsurgical resection of the inferior temporal gyrus. He has been seizure-free since the surgery. Patient 2 had an onset of complex partial seizures at the age of 18 due to right temporo-mesial cavernoma. Functional MRI studies identified a left hemisphere dominance for language. After conventional EEG monitoring, the patient was offered right anterior temporal lobectomy plus amygdalohippocampectomy including removal of the cavernoma. A suitable temporal craniotomy was performed, the dura was opened and the Sylvian fissure was identified. The prototype grid was placed on the temporal lobe for short-term (five minutes) recording. Wireless transmission of ECoG signals before the resection of cavernoma and the adjacent amygdala, hippocampus, and parahippocampal gyrus was conducted. The patient remains seizure-free six months after the procedure. Patient 3 had a history of developmental delay (IQ = 61) and schizophrenia; seizure onset was at 16 years of age. MRI was suggestive for left mesial temporal sclerosis. Video-EEG telemetry had captured typical seizures compatible with a left temporal lobe onset. Resective surgery was offered. The patient underwent temporal craniotomy and placement of the grid with five minutes of ECoG wireless transmission before undergoing left anterior temporal lobectomy and amygdalohippocampectomy. The patient has been seizure-free for four months at the time of this manuscript submission. In all three cases, the grid was easily placed on the brain and wireless ECoG transmission performed. The ECoG data were considered fully interpretable by the blinded expert (SS) and localization on interictal EEG abnormalities was concordant with that of the traditional grid and could have effectively directed the resection if used alone. The wireless ECoG quality was considered equivalent to that of traditional wired ECoG. In all three cases, wireless ECoG localized interictal spikes concordant with presurgical data and with subsequent wired recordings. No device-related complication was observed. 

## Discussion

After preliminary experience on non-human primates, a wireless device for ECoG recording in patients with drug-refractory epilepsy has been developed and tested on three patients undergoing invasive monitoring for ictal focus localization. The data presented here builds upon previous experiments in animals. The first prolonged recording with this chronic wireless ECoG device was obtained in a non-human primate, positioning a 16-channel grid over the sensorimotor cortex [3]. During this experiment, the impendence of each contact was found to remain substantially below 10 KΩ over six months, thus indicating the integrity of the signal acquisition. Wireless transmission of the ECoG signals maintained an excellent quality over time as well. Somatosensory evoked potentials were acquired and DCS was performed at set intervals as previously described [3,8]. Repeated somatotopic mapping of the primary motor cortex by direct cortical stimulation was also achieved successfully throughout the course of the experiment. Wireless recharge was provided by a custom-made recharging cage using a patented recharging system (patents EP2755469 B; US9831709 B2; 210 CN104427864 B). The recharging cage allowed a seamless recharge process without any additive stress to the primate who was allowed to move freely inside the cage throughout the entire duration of the experiment [3,7]. Removal of the device at the end of the 6 months monitoring period was remarkable for the total absence of adhesions between the grid and the cortex [3]. This experience encouraged us to develop a wireless device intended for clinical human applications, connected with a dedicated purpose-built 128 subdural grid, hosting 64 channels dedicated to monitoring and 64 channels to stimulation. Grid thickness is 0.5 mm with an electrode thickness of 25 µm. The grid can be manufactured in a range of sizes (44 by 35 mm, 66 by 55 mm or 91 by 60 mm), allowing the sampling of more restricted or wider regions of cortex. In this pilot human study, we used a 44 by 35 mm grid. The 128-channel configuration was chosen because it represents the best compromise between spatial sampling, associated data throughput and radio bandwidth used. Wireless data transfer exploits the recently introduced Medical Implant Communication System (MICS), a communication network that has been accepted worldwide for the transmission of diagnostic or therapeutic medical data designed to support uni- or bi-directional data transfer from medical devices implanted in the body to a programmer/controller outside [10]. MICS is characterized by a low-power, short-range (up to 2 meters), high-data-rate transmission with a core band of 402 to 405 MHz with good conductivity in the human body. The frequency used reduces the risk of interference with other devices and requires a transmission power limited to 25 microwatts. The programmer/controller unit can in his turn transmit the data received by the implanted device through existing communication networks, including the World Wide Web. With the implanted device, ECoG signals can be visualized in real-time by the treating physician on a smart-phone, tablet or dedicated laptop. The device is supplied by medical-grade certified batteries, needing a daily wireless recharge, which is made possible by the use of a PEEK-case allowing bidirectional wireless data transfer and recharge. Power transfer with battery recharge is obtained by electromagnetic coupling between the coil integrated on the implanted device and the one integrated on the wireless recharger. It is sufficient to place the wireless recharger above the skin overlying the PEEK case to obtain the electromagnetic coupling and start the power transfer. The wireless recharger provides visual and auditory signals: an auditory signal indicates that the coupling between the two coils was obtained, thus allowing the start of the recharge; a bicolor LED becomes green to signal a correct flow of energy and red if the coupling is lost and no recharge is happening. An internal microprocessor interrupts automatically the recharge phase if the temperature of the skin above the case increases by more than 2 °C, thus preventing skin burns due to excessive heating. The device can be implanted under the scalp for weeks or months, offering to patients undergoing invasive monitoring the advantage of prolonged ECoG recording without an increasing risk of infection. The added flexibility of the proposed device in terms of recording duration can enhance the probability of localizing the ictal-onset zone and extend the indication for invasive monitoring to a wider group of patients with drug-resistant epilepsy, which are today declined due to episodic seizures. These include cases with non-daily or infrequent seizures, which are not offered the option of invasive monitoring due to the limited chance of defining the ictal focus during the necessarily short recording time. The wireless device here described provides an enhanced diagnostic medium to localize neocortical ictal foci and can be used in adults and children, allowing early discharge and home monitoring through a dedicated Video-EEG station. Furthermore, long-term recordings can disclose seizure patterns not visible in the acute recordings done as an inpatient who has recently undergone surgery under general anesthesia. Prolonged EcoG recording may actually show rather surprising seizure patterns, including discordant lateralization of mesial temporal lobe epilepsy [10]. There is also evidence that stable ECoG recording may require up to 100 days to emerge after surgery [11]. Wireless transmission of ECoG recordings may in the future not only be of diagnostic value but also provide therapeutic options by closed-loop cortical stimulation. Closed-loop systems of neuromodulation may use wireless transmission of recordings from subdural grid electrodes to trigger or alter cortical stimulation using contacts on the grid itself. Remote sites of the central nervous system such as the thalamus and basal ganglia or the spinal cord can be reached by a wireless pairing of the intracranial contacts with, respectively, deep brain stimulation or spinal cord stimulation electrodes. Wireless ECoG recording is a promising technology for brain-computer interface (BCI) applications, with the potential to guide neuroprosthetic limbs or exoskeletons [3,8]. Wireless technology can also mitigate the need for tunneling of subcutaneous wires or extensions and therefore reduce the risk for skin erosion, dislocation, and infection.

## Conclusions

Short-term wireless ECoG monitoring was successfully performed in three patients undergoing epilepsy surgery. After this preliminary experience, we look forward to achieving prolonged wireless ECoG recording in selected patients. A fully implantable wireless ECoG device can extend substantially the recording time, thus enhancing the chances to achieve seizure focus localization while minimizing the risk of infections. Closed-loop stimulation and BCI applications are other promising applications of wireless ECoG. 
